# Association of Serum Uric Acid Concentration and Its Change with Cardiovascular Death and All-Cause Mortality

**DOI:** 10.1155/2020/7646384

**Published:** 2020-01-28

**Authors:** Ze-Xuan Dong, Ming Tian, Hua Li, Yang Wu, Xing-Guo Du, Jun-Wu Dong, Hui-Hui Xiao, Li-Ping Dong, Xiao-Hong Song

**Affiliations:** ^1^Shanxi Medical University, Jinzhong, 030600 Shanxi, China; ^2^Department of Nephrology, Wuhan Fourth Hospital, Puai Hospital, Tongji Medical College, Huazhong University of Science and Technology, Wuhan, 430030 Hubei, China; ^3^Department of Internal Medicine, JiangHan University, Wuhan, 430030 Hubei, China

## Abstract

**Objective:**

There is no consensus on the role of abnormal uric acid (UA) levels in the prognosis of patients undergoing hemodialysis. We therefore aimed to investigate the effects of changes in UA concentration on the risk of all-cause death and cardiac death in such patients.

**Method:**

In this retrospective cohort study, patients admitted to two hemodialysis centers performing maintenance hemodialysis (MHD) in Wuhan First Hospital and Fourth Hospital Hemodialysis Center from January 1, 2007, to October 31, 2017, were included.

**Results:**

In all, 325 patients undergoing MHD aged 59.7 ± 14.7 years, including 195 men (60%), were enrolled, with a median follow-up of 37 months. Serum UA (*p* < 0.001) was significantly higher in the surviving group than in the death group. No significant difference was found in UA variability (*p* < 0.001) was significantly higher in the surviving group than in the death group. No significant difference was found in UA variability (*p* < 0.001) was significantly higher in the surviving group than in the death group. No significant difference was found in UA variability (*p* < 0.001) was significantly higher in the surviving group than in the death group. No significant difference was found in UA variability (*p* < 0.001) was significantly higher in the surviving group than in the death group. No significant difference was found in UA variability (*p* < 0.001) was significantly higher in the surviving group than in the death group. No significant difference was found in UA variability (*p* < 0.001) was significantly higher in the surviving group than in the death group. No significant difference was found in UA variability (*p* < 0.001) was significantly higher in the surviving group than in the death group. No significant difference was found in UA variability (

**Conclusion:**

Low UA levels were closely related to all-cause mortality in patients undergoing MHD. Although UA levels had no significant effect on cardiac death, they had a good predictive value for long-term prognosis in patients on MHD.

## 1. Introduction

Studies have shown that hyperuricemia has a prevalence of more than 20% in the general population [1]. However, in patients with end-stage renal disease (ESRD), due to renal failure, glomerular filtration rate is severely reduced. The reabsorption of uric acid (UA) increases and secretion decreases in the proximal tubules. This causes a significant decrease in UA excretion, and excessive retention of UA in the body causes hyperuricemia [2]. Therefore, the incidence of hyperuricemia is higher in patients with ESRD, even more than 50% [3]. However, the average serum UA clearance per hemodialysis is about 1 g. Therefore, for hemodialysis patients, the UA level varies greatly [4].

It is well-known that vascular calcification, especially coronary artery calcification, is ubiquitous in patients undergoing hemodialysis. However, vascular calcification is an important cause of cardiovascular disease occurrence and death. The current etiology and pathogenesis of coronary calcification in patients with ESRD have not been fully elucidated. In recent years, many studies have shown that hyperuricemia is another common independent risk factor of vascular calcification, including dyslipidemia, smoking, hypertension, diabetes, and chronic kidney disease (CKD) [5, 6]. Higher levels of the serum UA led to wider coronary lesions, while heavier stenosis led to higher mortality rates [7–9]. Apart from vascular calcification, the effect of hyperuricemia on patients is also closely related to patients with hypertension, congestive heart failure, and atherosclerosis [10]. Therefore, Europe launched a consensus on the diagnosis and treatment of patients with high uric acidemia and high cardiovascular risk in 2018. It is mentioned in the consensus that serum UA should be controlled below 360 *μ*mol/L in all populations. However, for patients with a high risk of cardiovascular disease, serum UA should be controlled below 300 *μ*mol/L [11]. In fact, whether hyperuricemia is a risk factor for all-cause death and cardiac death in patients undergoing maintenance hemodialysis (MHD) has been a controversial topic. The early studies have found a “J”-shaped relationship between serum UA and all-cause mortality and cardiac mortality in patients with advanced CKD [12]. However, subsequent research has raised contradictory points: the increase in UA levels increased the risk of death in patients undergoing hemodialysis or peritoneal dialysis [13, 14]; in contrast, the lower levels of serum UA resulted in higher risks of all-cause death and cardiac death in patients undergoing hemodialysis [15, 16]. As time progresses, longitudinal increases in serum UA levels are associated with a reduction in all-cause mortality or cardiovascular death in these patients [4].

Therefore, the current evidence of UA level for all-cause death and cardiovascular death in hemodialysis patients is contradictory, and there is no consensus. This study is aimed at analyzing the effects of serum UA levels and their variability in all-cause death and cardiac death in patients undergoing MHD through a retrospective cohort study.

## 2. Materials and Methods

### 2.1. Participants

A retrospective cohort study was conducted to analyze the data of patients who underwent MHD in Wuhan First Hospital or Fourth Hospital Hemodialysis Center from January 1, 2007, to October 31, 2017. The inclusion criteria were as follows: (1) stable patients (dialysis vintage ≥ 12 months); (2) the initial dialysis mode was hemodialysis; (3) patients who underwent regular dialysis via their vascular access for 4 hours two or three times per week at a blood flow rate of 230-300 mL/min and a dialysis solution flow rate of 500 mL/min; the dialysis mode was not limited; (4) there were at least three serum UA tests per year, and the interval between two adjacent examinations was more than 2 months; for the frequent examination within 2 months, we took the average value; (5) the age of starting hemodialysis was ≥18 years old. The exclusion criteria were (1) patients with malignant tumor, (2) hemodialysis converted to peritoneal dialysis, and (3) combination of other serious organ diseases which affected the level of UA.

### 2.2. Method

Baseline clinical data were collected from patients who underwent hemodialysis, including those on primary renal disease, vascular access, hemoglobin, serum calcium phosphorus, albumin, UA, and parathyroid hormone (PTH). Blood specimens for biochemical tests were collected from the vascular access before midweek hemodialysis sessions. They were collected 7:00-8:00 h before morning sessions and 11:00-12:00 h before midday sessions. Patients were grouped according to the quartile of mean serum UA levels.

### 2.3. Endpoints of the Study

All patients were followed up from MHD treatment to the endpoint events (cardiac death or all-cause death), kidney transplantation, loss of follow-up, withdrawal from hemodialysis, or arrival of the study deadline (October 31, 2018). The endpoint event was all-cause mortality and cardiac death. Cardiac death was defined as death caused by ischemic heart disease, congestive heart failure, fatal arrhythmia, or other unexplained sudden death [17]. The calculation method for UA variability CV (coefficient of variance) is as follows: CV = (standard deviation/mean) × 100%, which can better reflect the degree of dispersion in the unit mean. This study was approved by the Ethics Committee of the First and Fourth Hospital of Wuhan and followed the ethical principles of the Declaration of Helsinki (http://www.wma.net/en/30publications/10policies/b3/index.html).

### 2.4. Statistics

Categorical and continuous variables are presented as percentage and mean ± SD, respectively, and nonnormal distributions are reported by the median concentration and interquartile range. The chi-square test and Wilcoxon rank sum test were used to compare categorical and continuous variables among groups, respectively. The analysis of linear trends was used to evaluate the association between increasing levels of UA and the risk of all-cause mortality or cardiac death after the patients were divided into quartiles based on the distribution of controls. A Kaplan-Meier analysis was used to evaluate the change in survival between the quartiles for UA, and the curves were compared using the log-rank test. A Cox proportional-hazards model, further adjusted in a stepwise manner for age, diabetes, vascular access, albumin, and UA variability, was used. A receiver operating characteristic (ROC) curve was used to evaluate the area under the curves (AUC) to identify UA with statistics used for comparing the different models. Differences with a *p* value of <0.05 (two-tailed) were considered to be statistically significant. The statistical software package R version 3.6.0 and SPSS for Windows, version 25.0 (SPSS, Chicago, IL, USA), were used for analysis. Graphs were constructed with GraphPad PRISM8 and Photoshop version 6.0.

## 3. Results

**Figure 1:**
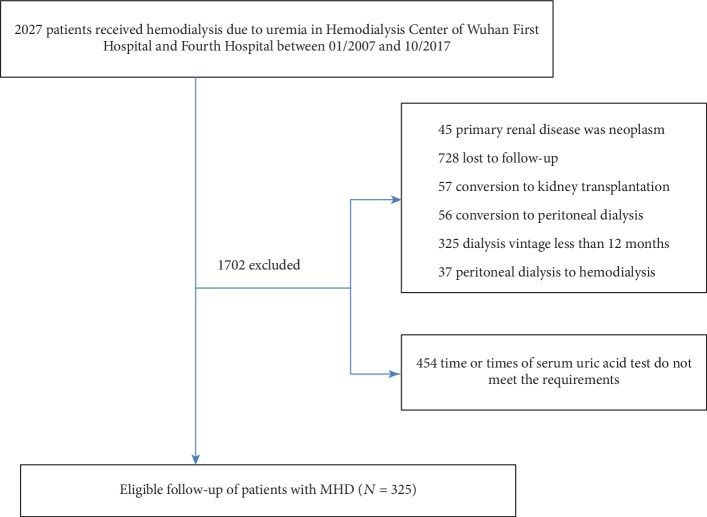
The flow chart of population selection. In total, 2027 patients were recruited from the Hemodialysis Center of Wuhan First Hospital and Fourth Hospital. After excluding 1702 participants, the final sample size of 325 participants was enrolled.

A total of 2027 patients were included in the study. According to the inclusion and exclusion criteria, the study eventually included 325 patients undergoing MHD (Figure 1).

Among the 325 patients who were enrolled, 195 patients were men (60%); 322 (99.1%) had hypertension. The average age of starting hemodialysis was 57 years, the youngest patient was 20 years old, and the oldest was 88 years old. The average dialysis vintage was 43 months. There were 236 patients (72.6%) who chose arteriovenous fistula (AvF) as the dialysis vascular access, and 89 patients (27.4%) were selected for dialysis with tunneled cuffed catheter. The primary disease of uremia included 26 cases (8%) who were with hypertensive renal damage, 3 (0.9%) had antineutrophil cytoplasmic antibody- (ANCA-) associated vasculitis with renal damage, 117 (36%) with primary glomerulonephritis, 147 (45.2%) with diabetic nephropathy, 11 (3.4%) with polycystic kidney disease, 3 (0.9%) with drug-induced renal damage, 10 (3.1%) with obstructive nephropathy, 6 (1.8%) with kidney transplant failure, and 2 (0.6%) had lupus nephritis. A total of 116 patients (35.7%) died at the end of follow-up; among them, 58 (17.8%) had cardiac deaths, accounting for 50% of all-cause deaths, including 24 who had ischemic heart disease, 8 died of malignant arrhythmia, 23 died of congestive heart failure, and 3 who had sudden cardiac death. Other causes of death include infections, gastrointestinal bleeding, multiple organ failure, and cerebrovascular diseases. In the 325 patients, serum UA detection times ranged from 4 to 53. To clarify the effects of different UA levels on all-cause death and cardiac death in patients undergoing hemodialysis, we performed further analysis based on the average serum UA level after quartile grouping.

Baseline clinical data of the four groups of patients are shown in Table 1. Compared with the other three groups, the patients in Quartile 1 were older at the age of starting hemodialysis. Compared with the other two groups, the proportion of AvF as vascular access was significantly higher in Quartile 3 and Quartile 4, at 76.5% and 82.9%, respectively. Although patients in all four groups had significantly different mean serum phosphorus and PTH concentrations, the values were still very close. The UA variability was significantly higher in Quartile 4 than in other quartiles. The primary diseases that caused uremia were diverse; however, the two primary diseases were mainly diabetes (147 cases, 45.2%) and primary glomerulonephritis (117 cases, 36%). Quartile 1 had the highest occurrence of all-cause death and cardiac death, at 55.6% and 30.9%, respectively.

We further compared the baseline clinical data between the patients who died and those who survived. The results showed that the age of starting hemodialysis of the death group was significantly higher than that of the surviving group (66.5 ± 12.0 vs. 55.9 ± 14.7, *p* < 0.001). The patients in the death group who used tunneled cuffed catheter were in a higher proportion, and serum phosphorus and PTH levels were better controlled in them than those in the surviving group. The mean serum UA level in the surviving group was significantly higher than that in the death group (403.3 ± 72.6 vs. 364.6 ± 79.1, *p* < 0.001). The primary disease of uremia in the surviving group was mainly primary glomerulonephritis (43.1%). However, in the death group, it was mainly diabetes (58.6%), which was much higher than that in the survival group (37.8%). We also found that there was no significant difference in UA variability between the two groups (0.3 ± 0.125 vs. 0.286 ± 0.136, *p* = 0.193), as shown in Table 2.

**Figure 2:**
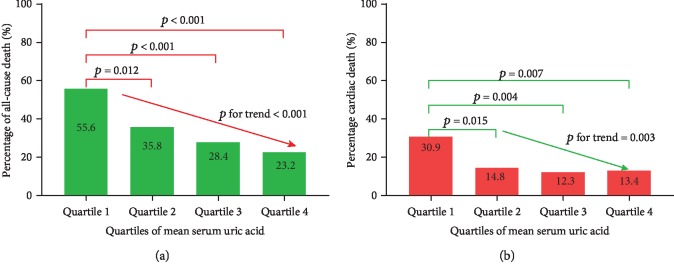
Mean serum uric acid (UA) and rates of (a) all-cause death and (b) cardiac death. Quartile 1, Quartile 2, Quartile 3, and Quartile 4 are quantile grouping based on UA levels.

**Figure 3:**
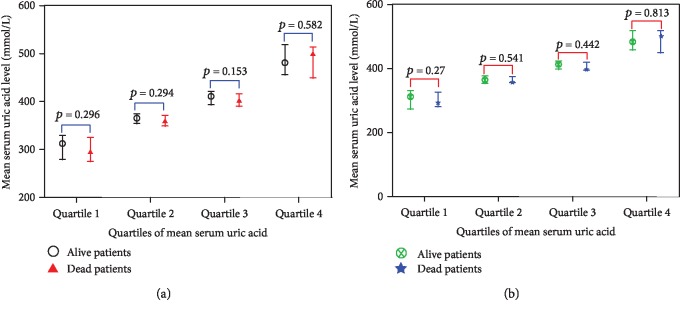
Comparing the differences in UA between dead and surviving patients in each group. Quartile 1, Quartile 2, Quartile 3, and Quartile 4 are quantile grouping based on UA levels.

When we did not consider the influence of dialysis vintage, uremic primary disease, and other factors, we found that the risk of all-cause death gradually decreased with the increase of UA among the four groups. Taking Quartile 1 as a reference, the following were observed: Quartile 2, OR = 0.4, 95% CI (0.2-0.8), *p* = 0.012; Quartile 3, OR = 0.3, 95% CI (0.2-0.6), *p* < 0.001; and Quartile 4, OR = 0.2, 95% CI (0.1-0.5), *p* < 0.001, *p* for trend < 0.001. The number of all-cause mortalities among the quartiles also gradually decreased; they were 45 (55.6%), 29 (35.8%), 23 (28.4%), and 19 patients (23.2%) in the four groups, respectively (Figure 2(a)). Cardiac death also showed the same trend, and with the increase of UA concentration, the risk of cardiac death was gradually reduced. Taking Quartile 1 as a reference, the following were observed: Quartile 2, OR = 0.4, 95% CI (0.2-0.8), *p* = 0.017; Quartile 3, OR = 0.3, 95% CI (0.1-0.7), *p* = 0.005; and Quartile 4, OR = 0.3, 95% CI (0.2-0.8), *p* = 0.009, *p* for trend = 0.003. The number of cardiac deaths among the four groups was 25 (30.9%), 12 (14.8%), 10 (12.3%), and 11 (13.4%), respectively (Figure 2(b)). Among the primary diseases of uremia in the enrolled patients, diabetes accounted for 45.2% and primary glomerulonephritis for 36% of them. Therefore, when adjusting for the patient's age, vascular access, diabetes, serum albumin, and UA variability, we found that this trend of influence only existed in all-cause death (*p* for trend = 0.001), while it did not exist for cardiac death (*p* for trend, 0.003-0.101) (Table 3). To determine whether there is a difference in UA levels between the death and survival groups, we further compared their UA concentrations. The results showed no significant difference (Figures 3(a) and 3(b)).

**Figure 4:**
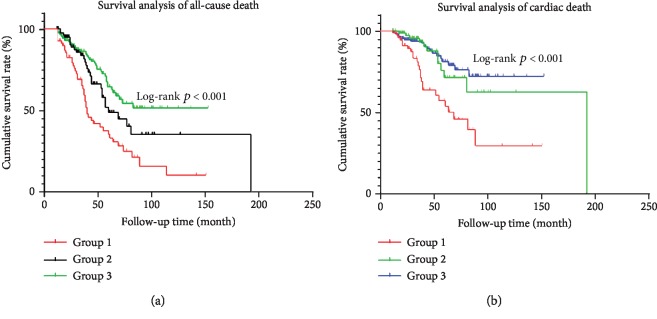
Kaplan-Meier curves for survival free of (a) all-cause death and (b) cardiac death stratified by groups based on the UA levels.

As it can be seen that there was no significant difference between Quartile 3 and Quartile 4 in all-cause death and cardiac death, we merged them into Group 3. Group 1 and Group 2 replaced Quartile 1 and Quartile 2, respectively. Then, we performed a single-factor Kaplan-Meier survival analysis. The results show that the median survival time for all-cause mortality (40 months, 95% CI, 33.261-46.739) and cardiac death (69 months, 95% CI, 43.608-94.392) in Group 1 was much lower than that in Group 2 and Group 3 (Log-rank *p* < 0.001), but the difference between Group 2 and Group 3 was not significant (*p* = 0.121 and *p* = 0.701, respectively) (Figure 4). The univariate Cox regression analysis showed that the age of starting hemodialysis, diabetes, serum albumin, vascular access and UA concentration and its variability interaction were risk factors for all-cause mortality. However, when adjusting for confounding factors, we found that an increase in UA levels reduced the risk of all-cause mortality, but the interaction between UA and variability no longer had this characteristic. In contrast, after adjusting for multiple confounding factors, both serum UA concentration and interaction between UA and its variability were not associated with cardiac death, as shown in Table 4.

**Figure 5:**
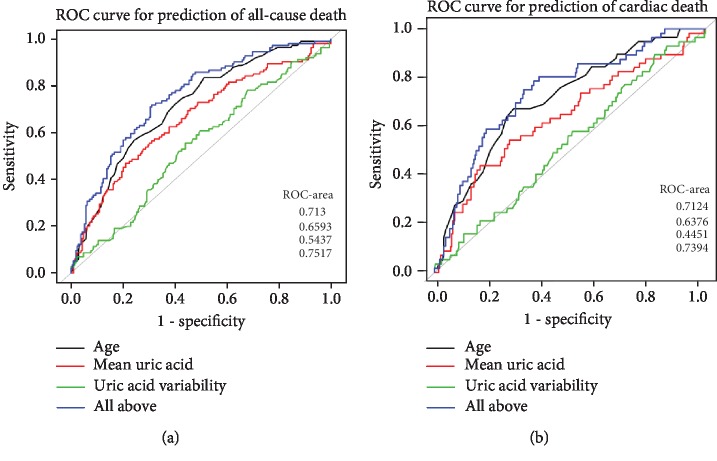
(a) Receiver operating characteristic curve (ROC) analysis for the prediction of all-cause death. Model 1 (black) includes age; Model 2 (red) includes UA; Model 3 (green) includes UA variability; and Model 4 (blue) includes age and UA and its variability. (b) ROC analysis for the prediction of cardiac death. Model 1 (black) includes age; Model 2 (red) includes UA; Model 3 (green) includes UA variability; and Model 4 (blue) includes age and UA and its variability.

To determine the predictive effect of the UA level and its variability on all-cause mortality and cardiac death in patients undergoing MHD, we used the ROC for analysis. The results showed that serum UA predicted all-cause mortality, the AUC was 0.6593 (95% CI, 0.596-0.7225), specificity was 78.95%, and sensitivity was 46.96%, while for cardiac death, AUC was 0.6376 (95% CI, 0.5529-0.7223). The variability of UA had less predictive value in all-cause mortality and cardiac death. Their AUC were 0.5437 and 0.4451, respectively. We found that the age of starting hemodialysis was an independent risk factor for all-cause mortality and cardiovascular death in patients undergoing MHD in both univariate and multivariate analyses. Therefore, we introduced it to predict all-cause death and cardiac death, and we found that there was no significant difference in the predictive value between age and UA (ROC-AUC ratio was 0.7134 vs. 0.6593, *p* = 0.2009; 0.7124 vs. 0.6376, *p* = 0.1888). However, when age was combined with UA and its variability, the diagnostic efficacy could be significantly improved. The AUC value for diagnosis of all-cause mortality was increased to 0.7517 (95% CI, 0.6969-0.8064, *p* = 0.0245), and the sensitivity was increased to 71.93%. The AUC value for diagnosis of cardiac death was increased to 0.7394 (95% CI, 0.6677-0.811, *p* = 0.2194) and the sensitivity was increased to 75% (Figures 5(a) and 5(b)).

## 4. Discussion

This article retrospectively analyzed the relationship between serum UA levels and its fluctuations in patients in terms of all-cause mortality and cardiac death. After adjusting for confounding factors, we found that long-term low-level UA (<339.491 *μ*mol/L) significantly increased the risk of all-cause mortality. However, the range of variation in UA levels could affect the occurrence of cardiovascular events in patients, which was not clear at this time. We also found that the higher age of starting hemodialysis of patients with serum UA levels increased the predictive value of long-term prognosis in these patients.

UA is the terminal metabolite of purine, and 80% of the total UA in the human body is produced by the catabolism of nuclear proteins and 20% produced by food which contains more purine. UA is basically present in the blood in the free form as monosodium urate. About 70% of UA is cleared by the kidneys, and the remaining 30% is cleared by the gastrointestinal tract [18]. Researchers had found that raised serum UA levels were significantly associated with a rapid decline in renal function and the incidence of CKD. Therefore, patients with chronic renal failure often develop hyperuricemia, especially in patients with advanced renal failure [19]. The biological role of UA in the human body is mainly reflected in two aspects. Firstly, the combination of UA with ammonia and urea plays an important role in the removal of nitrogen-containing compounds [20]. Secondly, UA is also an antioxidant. It can interact with hydrogen peroxide and hydroxyl radicals to effectively scavenge free radicals in the body, thus protecting vascular endothelial cells [21]. At present, there is no unified consensus on the impact of UA on the prognosis of patients undergoing hemodialysis. Some studies suggest that the raised UA levels increase the risk of death in hemodialysis or peritoneal dialysis patients [13, 14, 22]. Some researchers have also found that lower levels of UA increase cardiac death and all-cause death in these patients [23, 24]. Moreover, studies have shown that as time progresses, longitudinal increases in UA reduce all-cause mortality and cardiac death in these patients [25]. These follow-up studies were based on average UA or a certain baseline level of UA.

However, since the patient's UA level varies, the question remains whether the changed UA also affects the patient's prognosis. There is no similar study at the moment. We found that the UA variability has no effect on all-cause death and cardiac death. The patient's mean serum UA level was associated with prognosis. Without considering the confounding factors, all-cause death and cardiac death gradually decreased with the increase of UA, which is consistent with previous studies [25–27]. After adjusting for the confounding factors, the results of multivariate Cox regression analysis showed that with the increase of UA, all-cause death of patients still gradually decreased, but the impact on cardiac death was no longer obvious (*p* = 0.001, *p* = 0.058). Chang et al. and others recorded similar findings [24, 28]. We also found that average UA levels below 339.491 *μ*mol/L were an independent risk factor for all-cause mortality; this finding was consistent with that of a prospective cohort study by Korean researchers. Their study showed that a mean UA of <5.5 mg/dL in patients undergoing MHD was independently associated with all-cause mortality [29].

The mechanism of UA by which its level affects all-cause mortality and cardiovascular death in hemodialysis patients was unclear. It could essentially be the result of the comprehensive influence of many factors. High-risk deaths due to low UA levels may be related to the following causes. Studies have shown that UA levels were positively correlated with albumin and negatively correlated with the Charlson comorbidity index [30]. Thus, the risk of high mortality due to low UA levels might be because of bad nutritional status associated with hypoalbuminemia, and heavier comorbidities led to higher risk of death. Secondly, it could also be that excessive oxidative stress caused by low UA levels, and by inducing endothelial dysfunction, indirectly led to a higher risk of death [31, 32].

Our research found that whether it was single factor or multifactor analysis, the risk of all-cause death or cardiac death increased with age, which was consistent with previous studies [33, 34]. We used the ROC curve to analyze the predictive value of the age of starting hemodialysis for long-term prognosis in patients undergoing MHD. The results showed that there was no apparent difference in statistical efficacy compared with UA, but the combined predictive value of the two was significantly improved.

This study had several limitations. (1) This was a retrospective cohort study with a small sample size, which may have led to a bias. (2) The primary diagnosis of uremia in some patients was based on clinical diagnosis and not pathological diagnosis. (3) As we only collected data from patients at baseline, such as calcium, phosphorus, and PTH, we were unable to analyze the impact of these baseline changes on patient outcomes during follow-up. Therefore, we failed to correct the traditional risk factors for uremia and cardiovascular events during multivariate Cox regression analysis. (4) The oral medications commonly used by patients, cholesterol and Kt/v data, were not included in the analysis. (5) This study only specified the lower limit of UA level in patients undergoing MHD. Thus, the extent to which UA elevation requires intervention remains unclear.

In summary, through this retrospective cohort study, we showed that low UA levels are closely related to all-cause mortality in patients undergoing MHD. However, they have no significant effect on cardiac death. Moreover, they have a good predictive value for the long-term prognosis of such patients.

## Figures and Tables

**Table 1:** Characteristics of 325 patients with MHD by serum UA quartiles.

Range of uric acid	Quartile 1	Quartile 2	Quartile 3	Quartile 4	*p* value
	183.759-339.491	339.491-384.933	384.933-438.25	438.25-614.971	
	*N* = 81	*N* = 81	*N* = 81	*N* = 82	
Age (year)	63.8 ± 15.0	58.6 ± 14.6	59.5 ± 13.4	57.0 ± 15.2	0.026
Male (%)	41 (50.6%)	51 (63.0%)	51 (63.0%)	52 (63.4%)	0.266
Female (%)	40 (49.4%)	30 (37.0%)	30 (37.0%)	30 (36.6%)	
Dialysis vintage (months)	39.0 ± 28.3	44.3 ± 29.3	45.8 ± 25.9	44.7 ± 29.8	0.113
Tunneled cuffed catheter (%)	29 (35.8%)	27 (33.3%)	19 (23.5%)	14 (17.1%)	0.025
AvF (%)	52 (64.2%)	54 (66.7%)	62 (76.5%)	68 (82.9%)	
Hypertension (%)	80 (98.8%)	80 (98.8%)	80 (98.8%)	82 (100%)	0.796
Mean hemoglobin (g/L)	95.9 ± 13.7	97.4 ± 11.1	97.5 ± 12.8	97.3 ± 12.1	0.678
Mean phosphorus (mmol/L)	1.5 ± 0.4	1.7 ± 0.3	1.9 ± 0.4	1.8 ± 0.4	<0.001
Mean calcium (mmol/L)	2.2 ± 0.1	2.2 ± 0.1	2.2 ± 0.1	2.2 ± 0.1	0.675
Mean PTH (*μ*g/L)	277.7 ± 266.9	354.4 ± 291.3	412.9 ± 308.3	463.3 ± 345.2	<0.001
Mean albumin (g/L)	36.0 ± 4.0	37.6 ± 3.2	38.5 ± 2.8	39.0 ± 2.9	0.397
Mean serum uric acid (*μ*mol/L)	356.9 ± 72.6	375.7 ± 65.8	400.0 ± 75.4	426.7 ± 77.1	<0.001
Uric acid variability (%)	25.2 ± 14.5	28.1 ± 10.7	27.3 ± 10.7	37.0 ± 12.3	<0.001
Primary renal disease (*N*)					<0.001
Hypertensive kidney lesion	1 (1.2%)	8 (9.9%)	8 (9.9%)	9 (11.0%)	
ANCA	1 (1.2%)	0 (0.0%)	2 (2.5%)	0 (0.0%)	
Diabetes	57 (70.4%)	40 (49.4%)	30 (37.0%)	20 (24.4%)	
Glomerulonephritis	17 (21.0%)	24 (29.6%)	31 (38.3%)	45 (54.9%)	
Drug-induced kidney damage	1 (1.2%)	0 (0.0%)	1 (1.2%)	1 (1.2%)	
Polycystic kidney	2 (2.5%)	4 (4.9%)	2 (2.5%)	3 (3.7%)	
Renal allograft dysfunction	1 (1.2%)	1 (1.2%)	2 (2.5%)	2 (2.4%)	
Lupus nephritis	0 (0.0%)	0 (0.0%)	1 (1.2%)	1 (1.2%)	
Obstructive nephropathy	1 (1.2%)	4 (4.9%)	4 (4.9%)	1 (1.2%)	
Outcome					
All-cause death	45 (55.6%)	29 (35.8%)	23 (28.4%)	19 (23.2%)	<0.001
Cardiac death	25 (30.9%)	12 (14.8%)	10 (12.3%)	11 (13.4%)	0.005

AvF = arteriovenous fistula; PTH = parathyroid hormone; *N* = number; ANCA = antineutrophil cytoplasmic antibodies; MHD = maintenance hemodialysis; UA = uric acid.

**Table 2:** Characteristics of alive and dead patients.

Status	Alive	Dead	*p* value
Number of patients	*n* = 209	*n* = 116	
Range of uric acid variability values (%)	30 ± 12.5	28.6 ± 13.6	0.193
Age (year)	55.9 ± 14.7	66.5 ± 12.0	<0.001
Male	124 (59.3%)	71 (61.2%)	0.741
Female	85 (40.7%)	45 (38.8%)	
Tunneled cuffed catheter (%)	50 (23.9%)	39 (33.6%)	0.06
AvF (%)	159 (76.1%)	77 (66.4%)	
Hypertension (%)	207 (99.0%)	115 (99.1%)	0.932
Hemoglobin (g/L)	97.8 ± 12.4	95.7 ± 12.4	0.132
Phosphorus (mmol/L)	1.8 ± 0.4	1.6 ± 0.4	<0.001
Calcium (mmol/L)	2.2 ± 0.2	2.2 ± 0.1	0.904
PTH (*μ*g/L)	428.4 ± 334.9	277.9 ± 225.0	<0.001
Albumin (g/L)	38.2 ± 3.1	36.8 ± 3.8	<0.001
Uric acid (*μ*mol/L)	403.3 ± 72.6	364.6 ± 79.1	<0.001
Primary renal disease (*N*)			0.003
Hypertensive kidney lesion	16 (7.7%)	10 (8.6%)	
ANCA	1 (0.5%)	2 (1.7%)	
Diabetes	79 (37.8%)	68 (58.6%)	
Glomerulonephritis	90 (43.1%)	27 (23.3%)	
Drug-induced kidney damage	3 (1.4%)	0 (0.0%)	
Polycystic kidney	7 (3.3%)	4 (3.4%)	
Renal allograft dysfunction	6 (2.9%)	0 (0.0%)	
Lupus nephritis	2 (1.0%)	0 (0.0%)	
Obstructive nephropathy	5 (2.4%)	5 (4.3%)	

AvF = arteriovenous fistula; PTH = parathyroid hormone; *N* = number; ANCA = antineutrophil cytoplasmic antibodies.

**Table 3:** The association between mean uric acid levels in hemodialysis patients and the risk of all-cause mortality.

The association between mean uric acid levels in hemodialysis patients and the risk of all-cause mortality					
Variable	Quartile 1 (183.759-339.491 *μ*mol/L) Reference	Quartile 2 (339.491-384.933 *μ*mol/L) OR (95% CI)	Quartile 3 (384.933-438.25 *μ*mol/L) OR (95% CI)	Quartile 4 (438.25-614.971 *μ*mol/L) OR (95% CI)	*p* value for trend
Model 1	1	0.4 (0.2-0.8)	0.3 (0.2-0.6)	0.2 (0.1-0.5)	<0.001
Model 2	1	0.6 (0.3-1.1)	0.4 (0.2-0.7)	0.3 (0.1-0.7)	0.001
The association between mean uric acid levels in hemodialysis patients and the risk of cardiac death					
Variable	Quartile 1 (183.759-339.491 *μ*mol/L) Reference	Quartile 2 (339.491-384.933 *μ*mol/L) OR (95% CI)	Quartile 3 (384.933-438.25 *μ*mol/L) OR (95% CI)	Quartile 4 (438.25-614.971 *μ*mol/L) OR (95% CI)	*p* value for trend
Model 1	1	0.4 (0.2-0.8)	0.3 (0.1-0.7)	0.3 (0.2-0.8)	0.003
Model 2	1	0.5 (0.2-1.2)	0.4 (0.2-1.0)	0.6 (0.2-1.4)	0.101

Model 1: unadjusted. Model 2: adjusted for age, vascular access, albumin, diabetes, and uric acid variability.

**Table 4:** Cox regression analysis of all-cause mortality and cardiac death.

Variable	Univariable		Multivariable	
	HR (95% CI)	*p* value	HR (95% CI)	*p* value
Cox regression analysis of all-cause mortality				
Age (years)	1.058 (1.042-1.074)	<0.001	1.044 (1.026-1.062)	<0.001
Diabetes (yes = 1, no = 0)	2.154 (1.477-3.14)	<0.001	1.751 (1.138-2.696)	0.011
Vascular access (1 = catheter, 2 = AvF)	0.406 (0.274-0.602)	<0.001	0.759 (0.484-1.189)	0.228
Albumin (g/L)	0.819 (0.776-0.865)	<0.001	0.899 (0.842-0.96)	0.001
Mean uric acid (*μ*mol/L)	0.995 (0.992-0.997)	<0.001	0.996 (0.992-1.000)	0.035
Mean uric acid^∗^ variability	1.0 (1.0-1.0)	0.042	1.0 (1.0-1.0)	0.223
Cox regression analysis of cardiac death				
Age (years)	1.073 (1.049-1.098)	<0.001	1.066 (1.037-1.096)	<0.001
Diabetes (yes = 1, no = 0)	3.157 (1.788-5.572)	<0.001	3.114 (1.577-6.150)	0.001
Vascular access (1 = catheter, 2 = AvF)	0.322 (0.187-0.554)	<0.001	0.605 (0.324-1.130)	0.115
Albumin (g/L)	0.835 (0.77-0.905)	<0.001	0.957 (0.869-1.054)	0.369
Mean uric acid (*μ*mol/L)	0.994 (0.99-0.998)	0.001	0.994 (0.988-1.0)	0.056
Mean uric acid^∗^ variability	1.0 (1.0-1.0)	0.146	1.0 (1.0-1.0)	0.098

HR (hazard ratio) indicates the relative increased risk of all-cause or cardiac death with each change in age, mean uric acid, uric acid variability, albumin, diabetes positive, or vascular access. Mean uric acid^∗^variability reflected the interaction between uric acid and its variability. In multivariable regression analysis, these six variables were included in the same model.

## Data Availability

The data used to support the findings of this study are included within the article.
